# Super Hydrophilic Activated Carbon Decorated Nanopolymer Foam for Scalable, Energy Efficient Photothermal Steam Generation, as an Effective Desalination System

**DOI:** 10.3390/nano10122510

**Published:** 2020-12-14

**Authors:** Naila Arshad, Iftikhar Ahmed, Muhammad Sultan Irshad, Hong Rong Li, Xianbao Wang, Shafiq Ahmad, Mohamed Sharaf, Muhammad Firdausi, Mazen Zaindin, Muhammad Atif

**Affiliations:** 1 Institute of Quantum Optics and Quantum Information, School of Physics, Xi’an Jiaotong University, Xi’an 710049, China; nailasehar371@gmail.com (N.A.); hrli@xjtu.edu.cn (H.R.L.); 2 Energy Research Centre, COMSATS University, Islamabad, Lahore Campus 54000, Pakistan; dr.iftikhar.Ahmed@live.fr (I.A.); wangxb68@aliyun.com (X.W.); 3 Ministry-of-Education Key Laboratory for the Green Preparation and Application of Functional Materials, Hubei Key Laboratory of Polymer Materials, School of Materials Science and Engineering, Hubei University, Wuhan 430062, China; sultan.danish93@gmail.com; 4 Industrial Engineering Department, College of Engineering, King Saud University, P.O. Box 800, Riyadh 11421, Saudi Arabia; mfsharaf@ksu.edu.sa (M.S.); 438106660@student.ksu.edu.sa (M.F.); 5 Department of Statistics and Operations Research, College of Science, King Saud University, P.O. Box 800, Riyadh 11421, Saudi Arabia; zaindin@ksu.edu.sa; 6 Department of Physics and Astronomy, College of Science, King Saud University, Riyadh 11451, Saudi Arabia; muhatif@ksu.edu.sa

**Keywords:** activated carbon, melamine nanofoam, nano-desalination, photothermal steam

## Abstract

Clean water scarcity is still an intense, prolonged global issue that needs to be resolved urgently. The solar steam generation has shown great potential with a high energy conversion efficiency for clean water production from seawater and wastewater. However, the high evaporation rate of water cannot be preserved due to the inevitable fouling of solar absorbers. Herein, a self-floatable and super hydrophilic solar-driven steam generator composed of activated carbon coated melamine foam (ACM). The deposited ACM photothermal layer exhibits outstanding solar absorption (92%) and an efficient evaporation rate of 1.27 kg m^−2^ h^−1^, along with excellent photothermal conversion efficiency (80%) as compared to commercially available primitive solar stills. The open porous assembly of melamine foam equipped with 80% flexibility (0.8 MPa) enabled smooth water transport and sustain heat accumulation within the matrix. The thermal insulation of ACM is 10 times greater than pure water. Moreover, open porous assembly of designed solar-powered steam generator rejects salt ions as well as volatile organic compounds efficiently. The low-cost and facile fabrication of photothermal based water production presents a potential solution to single step drinking water supply from various resources of the sea, the lakes and mixtures of emulsified oil and industrial wastewater.

## 1. Introduction

The severe energy crisis and water scarcity have become a global challenge for all humankind over time. Renewable energy resources play an essential role to meet these challenges, especially solar energy. Solar thermal technology is a direct means to harvest maximum solar energy in a wide range of heating and energy storage applications [1,2,3,4]. One of the most attractive approaches regarding solar harvesting is solar-driven evaporation, which comprises the generation of vapors below a boiling temperature and steam production. It is an admitted fact that solar heat can be absorbed efficiently at the water-air interface and accumulate. This accumulated heat could be localized in a heat-insulating matrix. The localization of heat leads towards maximum solar-driven steam generation as compared to other traditional low yield water evaporation (due to the vast loss of solar heat to the bulk water and surrounding) [5,6,7]. In recent years, many successful attempts were accomplished regarding photothermal conversion-based steam generation without any input energy in various fields such as clean water production, photothermal therapy, photoimaging, energy generation, and many other industrial applications [5,8,9,10,11,12,13,14,15]. Solar-driven interfacial evaporation is one of the most promising alternatives to conventional desalination technologies in recent years. Unfortunately, it remains a significant challenge to integrate all desired functions in a single photothermal system. Despite the potential improvements from coupling solar evaporation structures, several unresolved challenges remain. Among these challenges are: Salt accumulation in the solar evaporation structure under continuous evaporation, stable evaporation rate during condensation, heat losses under real seawater conditions, shrinking costs of condensing infrastructure [16,17]. Based on these merits, tremendous efforts have been implored to find out excellent photothermal materials with the incorporation of unique characteristics, e.g., ultra-band light absorption, super hydrophilicity, mechanical robustness, porosity, and thermal insulation, etc. [2,18,19,20].

Activated carbon (AC) exhibits high surface to volume ratio owing to its small size and can absorb a wide range of solar spectrum due to its closely packed energy level of loosely π electrons due to weak Van Dar Waals forces, to excite electrons and relax to their ground states in the form of heat generation [2,21,22]. Prepared solar harvesting device shows good absorption (UV–VIS), super hydrophilicity, refractive index (~2.4), and outstanding stability in water. In 2017, Li at el. reported the fabrication of activated carbon fiber for solar steam generation with a solar conversion rate of 1.22 kg m^−2^ h^−1^ and output efficiency of 79.4% under one sun [23]. However, the AC fiber shows efficient evaporation efficiency but complex fabrication process. The proposed system adopts low-cost and facile fabrication of steam generation device to meet the desired mechanical strength. Indeed, the open porous assembly of water transport channels may lead towards long term efficacy on a commercial scale to avoid weak, fragile nature of devices [2,24,25,26,27].

**Figure 1:**
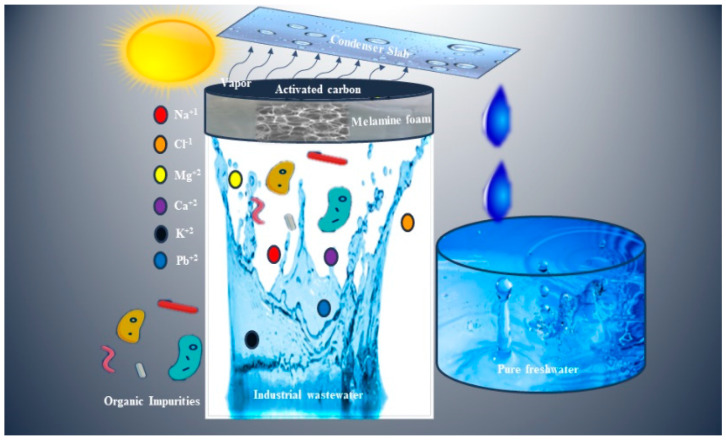
Schematic illustration of activated carbon decorated melamine foam-based steam generator.

In this contribution, a self-floating and super hydrophilic activated carbon decorated melamine foam (ACM) based solar-driven evaporation system is designed for clean water generation. The presented work focused on the mechanically robust solar-driven evaporation system that filtered volatile organic compounds (VOCs), seawater desalination, wastewater treatment. The ACM based solar-driven evaporation system equipped with tremendous light absorption (92%), self-floating ability (lightweight), efficient evaporation rate (1.27 kg m^−2^ h^−1^) under one sun, which ensures its portability and durability. The system exhibits the lowest thermal conductivity (0.06262 W m^−1^ K^−1^), which is far less than that of standard water (0.6 W m^−1^ K^−1^). The ACM attains natural super hydrophilicity due to its extraordinary open porous surface that gives maximum evaporation along with outstanding rejection ability. The most significant characteristic of the ACM photothermal system is to filter out the freshwater from both organic and inorganic impurities contaminated source water. Mainly, the real-time investigation was successfully employed to yield freshwater from wastewater, showing the ability to filter out heavy toxic heavy metals from lake wastewater as illustrated in Figure 1.

## 2. Materials and Methods

### 2.1. Materials

The activated carbon pellets were purchased from Beijing Blue Forest Carbon Industry Co., Ltd. (Beijing, China). The volatile binder terpineol (C_10_H_18_O), and absolute ethanol were bought from Aladdin Industrial Corporation (Shanghai, China). The desired AC pellets derived from raw coal that was milled efficiently. The obtained powder was made adhesive and pressurized into fine slurry. Further, the slurry was molded and carbonized at high temperature. Final, the carbonated AC was passes through steam activation process. The detailed synthesis procedure was carried out by Beijing Blue Forest Carbon Industry Co., Ltd. Similar approach of biomass based activated carbon has been reported [28,29]. Melamine sponge was bought from Shanghai Jinshang Auto Parts Co., Ltd (Shanghai, China). All the purchased chemicals were well meeting to 99% purity level and were employed for material fabrication without any further purification process.

### 2.2. Fabrication of ACM Solar Steam Generator

In order to collect fine activate carbon powder (AC), the activated carbon pellets were grinded via electronic crushing machine and then ball milling for three hours. The obtained fine and odorless AC powder were further grinded using mortar pestle and stored for next procedure. Melamine foam was specified as substrate owing to its super hydrophilic and self-floating nature and prepared as a cylindrical shape (2 cm × 2 cm × 3 cm = 12 cm^3^). However, 1.2 g activated carbon powder was mixed into 1 mL volatile binder terpineol (C_10_H_18_O) using mortar pestle for making a slurry for coating. Afterward, the obtained AC black gel was evenly coated on prepared cylindrical shaped melamine foam using glass roller. Subsequently, the AC coated melamine foam-based device was heated up to 120 °C in a microwave oven for 50 min to depart terpineol. Finally, an activated carbon decorated melamine foam-based steam evaporator was characterized via different spectroscopies to investigate its aptitude as a solar-driven steam generation device.

### 2.3. Solar-Driven Setup

Steam generation experiments were carried out by a solar simulator (PLS-FX300HU, Beijing Perfect Light Technology Co., Ltd., Beijing, China) outputting a flux of 1 kW m^−2^ (one sun). An optical filter was used to achieve a regular 1.5 G AM spectrum. The fabricated ACM steam generating device with an approximate thickness of 4 cm was set solar beam spot under one sun intensity by floating it on a beaker filled with pure water (or simulated seawater for desalination tests). An electronic analytical balance (Mettler Toledo, ME204, Across International, Sparks, NJ, USA) was employed to compute the mass variation with a resolution of 0.001 g. After 40 min when the whole system is stabilized, the evaporation rate of the system was measured under one solar flux. A thermal infrared image camera (FLIR E4 Pro, Phase 1 Technology Corp., Deer Park, NY, USA) was utilized for recording the ACM surface temperature along with two temperature-sensing thermocouples mounted in the sample top and bottom surface, respectively. By employing an inductively coupled plasma-optical emission spectrometry (ICP-OES, EP Optimal 8000, Perkin Elmer, San Jose, CA, USA), condensing water, saline water ion concentrations were calculated. The whole experimental process was conducted under ambient conditions, at temperature (~25 °C) and humidity ~48%. Mass variations for ACM were computed through an electronic analytical balance (0.01 g inaccuracy) under one solar flux. Utilizing a hand-held optical meter, along with thermocouples, the surface temperature was regulated.

### 2.4. Preparation Volatile Organic Compounds (VOCs)

Volatile organic compounds (VOCs) are remaining a significant challenge in desalination systems, where inorganic ions could be easily ejected. To cope this issue, several VOCs emulsions were prepared to examine the elimination capability of activated carbon decorated melamine foam (ACM) based solar-driven evaporation system from contaminated water to obtain freshwater. These emulsions are surfactant stabilized such as ethanol/water, formaldehyde/water, toluene/water mixtures (*v*/*v* = 1:1) were prepared, respectively. Moreover, muddy wastewater was also treated via ACM based steam-based generation and obtained from various sources to check the potential for real time application of super hydrophilic and self-floating system to crop freshwater for remote sensing areas.

### 2.5. Material Characterization

The morphology was characterized by a field emission scanning electron microscope (FESEM, JSM7100F, Shimadu, Kyoto, Japan). Sample phase structural analyzation was conducted via X-ray diffraction (XRD, Bruker D8 phaser, Bruker, Coventry, UK) sourced by Cu Kα with operating potential of 40 kV and current 200 mA. By employing ultraviolet-visible (UV–VIS) spectroscopy (Shimadu UV−VIS−NIR UV-3600 double beam spectrophotometer) along with an integrating sphere, photonic transmittance (T) and reflectance (R) were calculated in range of 250 to 2500 nm. Light absorption (A) was computed via A = 1 − T − R. X-ray photoelectron spectroscopy (XPS) (Escalab 250Xi, Thermo Fisher Scientific, Waltham, MA, USA) was used to compute the element configuration sourced by monochromatic X-ray Mg Ka radiation.

## 3. Results and Discussion

### 3.1. Open Porous Assembly

**Figure 2:**
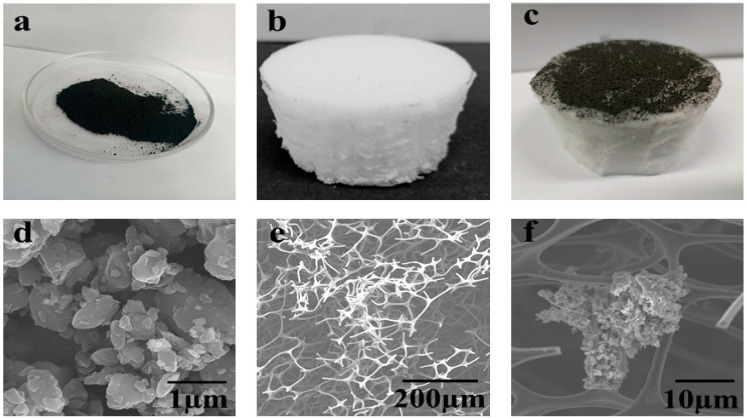
Surface morphologies and microstructure (**a**–**c**) complete fabrication of ACM solar evaporator from activated carbon (**a**) pure activated carbon (AC); (**b**) cylindrical shaped melamine foam; (**c**) activated carbon decorated melamine (ACM) foam-based steam generator. FESEM images labelled (**d**–**f**) as (**d**) homogeneous non-uniform sized particles; (**e**) porous structure of melamine foam; (**f**) activated carbon clusters over microchannels of melamine foam.

The microstructure and morphology of pure activated carbon powder, natural super hydrophilic melamine polymer foam, and activated carbon decorated melamine foam (ACM) were carried out using field emission electron microscopy (FESEM). The open porous structure allows smooth water transport and avoid blocking of heavy metals ions or salt ions that restrict the continuous evaporation rate. Indeed, numerous investigations were examined recently to develop a well-aligned open porous structure instead of randomly-oriented pores. Figure 2a represents the super black fine and odorless powder of activated carbon granules less than 0.1 mm size. Figure 2b shows the cylindrically-shaped (2 cm × 2 cm × 3 cm = 12 cm^3^) engineered melamine foam. As compared to other polymer foams, the melamine foam exhibits good heat insulating ability as well as excellent orientation of pores. Moreover, open porous surface of melamine foam also provides adhesion property for any photothermal layer without any surface degradation [28]. The final product activated carbon decorated melamine foam represented in Figure 2c. The surface morphology of activated carbon powder is shown in Figure 2d that depict its homogeneous non-uniform sized particles. Figure 2e represents FESEM image of the porous structure of melamine foam. The porosity of melamine foam structure is seen clearly depicting its super hydrophilic nature. These microchannels affirms the open porous structure and allows smooth transportation of water through melamine foam. Figure 2c presents the deposition of activated carbon clusters over melamine foam which is entangled in the microchannels of melamine foam. Deposition of carbon over the melamine foam surface makes it absorb high amounts of solar energy, which, in turn, increases the evaporation efficiency.

### 3.2. Structural and Chemical State of ACM

**Figure 3:**
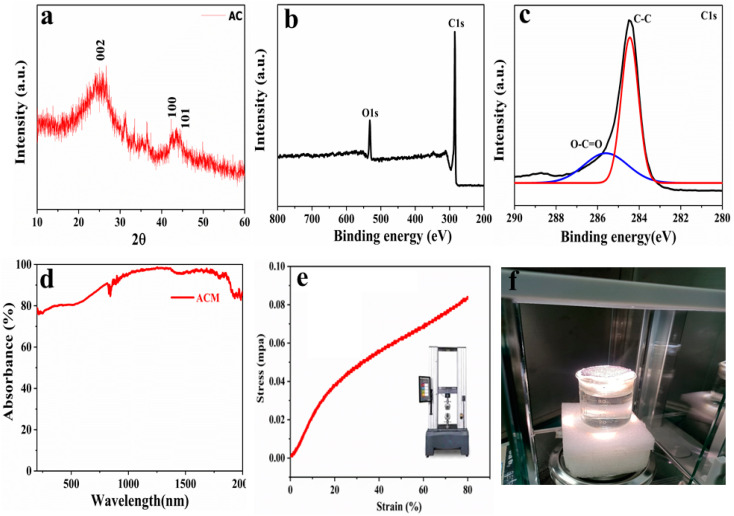
Crystallographic analysis of pure activated carbon by X-ray diffraction and X-ray photoemission spectroscopies; (**a**) XRD pattern of pure activated carbon powder; (**b**) XPS survey of AC; (**c**) C1s spectra of AC that reveals oxygen presence; (**d**) wide range of UV–VIS absorption of AC; (**e**) ultimate tensile stress curve of ACM evaporator; (**f**) real-time demonstration of ACM evaporator during steam generation.

The crystallographic structural phase of activated carbon is demonstrated in Figure 3a. The obtained X-ray diffraction pattern is well matched with the standard JCPDS file (41–1487), predicting the presence crystallinity in structure. The observed spectra contain three main peaks that are indexed at (002), (100), and (101) indicating the presence of crystal planes. The appearance of sharp peak near 26° reveals the main crystalline peak of AC exhibiting the highly aligned symmetry of disordered carbon layer forming turbo static structure deposited over melamine foam [30,31]. The chemical state of activated carbon powder was investigated using X-ray photoemission spectroscopy (XPS), as illustrated in Figure 3b. The XPS survey reveals two major peaks of activated carbon material that corresponds to carbon and oxygen. These two peaks clearly define the purity of activated carbon and there is no other species present on the surface of activated carbon. The C1s spectra of activated carbon also indicates two shoulder peaks that relate to C–C, and O–C=O bonds at 284.7 eV, and 286.3 eV, respectively. The O–C=O signals reveals the excessive concentration of oxygen species present over the surface of activated carbon. The presence of excessive oxygen could be beneficial for photothermal conversion behavior due to its closely packed energy level of loosely π electrons due to weak Van Dar Waals forces, to excite electrons and relax to their ground states in the form of heat generation [2,29]. Figure 3d represents the wide range of solar absorption spectra of activated carbon that shows outstanding solar absorption (92%) that is calculated using (1 − T − R). The excellent solar absorption of AC enabled it to convert solar energy into thermal energy that could be utilized for steam generation. Melamine foam owes naturally open porous structure and promote super hydrophilicity. The open porous structure also sustains its extreme flexibility. ACM evaporators possess its elastic nature under relaxation. Moreover, ultimate tensile stress (UTS) experiment was carried out to evaluate its mechanical robustness, as illustrated in Figure 3e. The observed results reveal its 80% compressive stain without any surface degradation or material deterioration. This significant flexibility factor enhanced its long-term efficacy and portability of the device. Figure 3f demonstrates the real-time ACM solar evaporator during water evaporation investigation.

### 3.3. ACM Based Steam Generator

**Figure 4:**
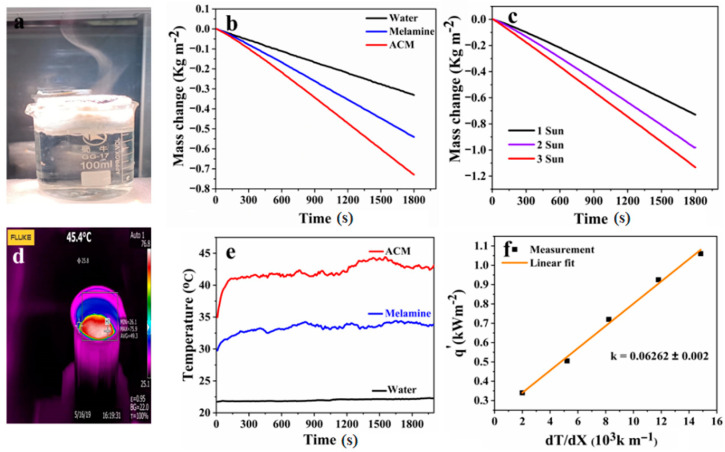
Solar steam generation investigations; (**a**) real-time demonstration of activated carbon decorated melamine foam (ACM) based steam generation; (**b**) mass change profile of three different systems; (**c**) mass change profile of ACM steam generator under different solar intensity; (**d**) I.R. image of ACM during functioning; (**e**) surface temperature of three different systems; (**f**) thermal conductivity of ACM device under wet state.

Super hydrophilic and versatile activated carbon decorated melamine foam (ACM) steam generator equipped with 92% absorbing capacity is manifested as an efficient solar-driven steam generating system. Thermal management is an effective factor in increasing the overall effectiveness of the solar-driven evaporation system. Activated carbon coated melamine foam exhibits excellent thermal insulation and could establish effective heat accumulation for efficient solar steam generating device, as illustrated in Figure 4a. We have computed a comparative analysis of photothermal conversion process regarding three different systems, including pure water, simple melamine foam, and activated carbon decorated melamine foam (ACM). For these three systems, time-dependent mass loss was computed under one sun intensity (1 kW m^−2^) using weighing balance. After irradiating the systems for one hour, ACM depicts the maximum weight loss comparative to water and melamine as shown by Figure 4b. By employing ACM, weight losses were computed under different irradiating intensities up to 3 kW m^−2^, indicating that by increasing the illumination intensity, greater weight loss (1.82 kg m^−2^) is detected using ACM solar evaporator as illustrated in Figure 4c. The water transport through these well oriented microchannels pathways, and thermal insulation potential was inspected using thermal image captured by an infrared camera (FLIR E8, USA). Figure 4d represents the I.R. surface image of the ACM, which demonstrates the heat aggregation within insulating matrix and obtained a 45.4 °C temperature under 1 kW m^−2^. The I.R. recorded temperature was confirmed using keithley source measurement unit along with thermocouples, as illustrated in Figure 4e. The observed surface temperature of pure water, simple melamine foam and ACM solar evaporator, which turn out to be highest in the presence of ACM. The thermal properties were computed for solar driven evaporating system using thermal conductivity meter (Hot Disk, TPS 2500, Sweden Hot Disk Cooperation, Gothenburg, Sweden). As demonstrated by Figure 4f, ACM reveals the minimum thermal conductivity 0.06262 ± 0.002 W m^−1^ K^−1^, which is approximately 10 times less than that of water (0.6 W m^−1^ K^−1^).

### 3.4. Evaporation Efficiency and VOCs Rejection

**Figure 5:**
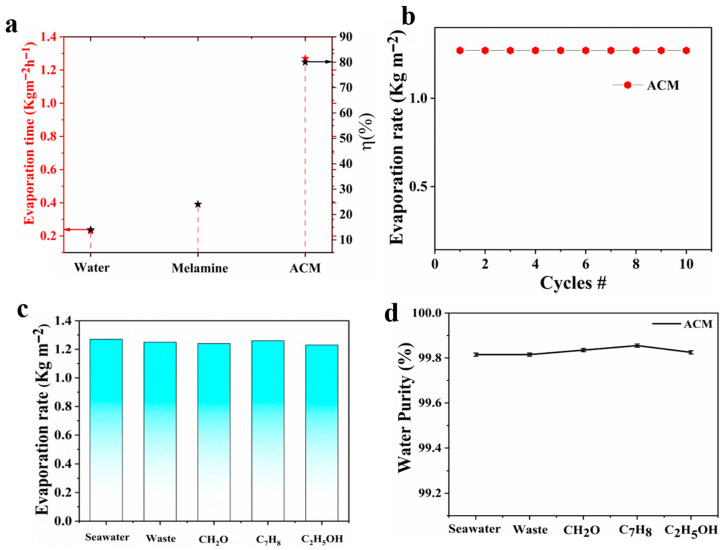
Photothermal conversion efficiencies and nano filtering of volatile organic compounds (VOCs): (**a**) evaporation rate and photothermal conversion efficiencies of three different systems; (**b**) consecutive solar-driven steam generation cycles; (**c**) smooth evaporation rate using ACM steam generator against different VOCs emulsions; (**d**) water purity profile of condensed water from different VOCs emulsions.

A comparative analysis for evaporation rates was computed for three systems including water, Melamine foam and ACM as demonstrated in Figure 5a. It is obvious from graph that evaporation rate of ACM solar steam generator (1.27 kg m^−2^ h^−1^) is higher comparative to melamine foam and water along with excellent photothermal conversion efficiency of 80%. The ACM is an effective and stable solar driven steam generating device without any fluctuation in evaporation rate under consecutive ten cycles and withstand evaporation rate 1.27 kg m^−2^ h^−1^ as depicted from Figure 5b. The smooth evaporation efficiency illustrates the outstanding mechanical flexibility of ACM that portrays its long-term efficacy and portability. Moreover, source water was mixed with different volatile organic impurities and evaporation rate was computed including CH_2_O, C_7_H_8_, and C_2_H_5_OH, respectively. The observed evaporation rates affirm that there is no significant change in evaporation rate under volatile organic compounds (VOCs) based impurities as compared to ordinary wastewater, as shown in Figure 5c. These VOCs are still remaining a prolong issue in water treatment process. Figure 5d affirms the enormous rejection up to 99.8% from VOCs contained water and collected water meet the standard of drinking quality. The obtained results open new window for photocatalysis based recovery of precious metals and production of fuel gas.

### 3.5. Desalination and Water Recovery

**Figure 6:**
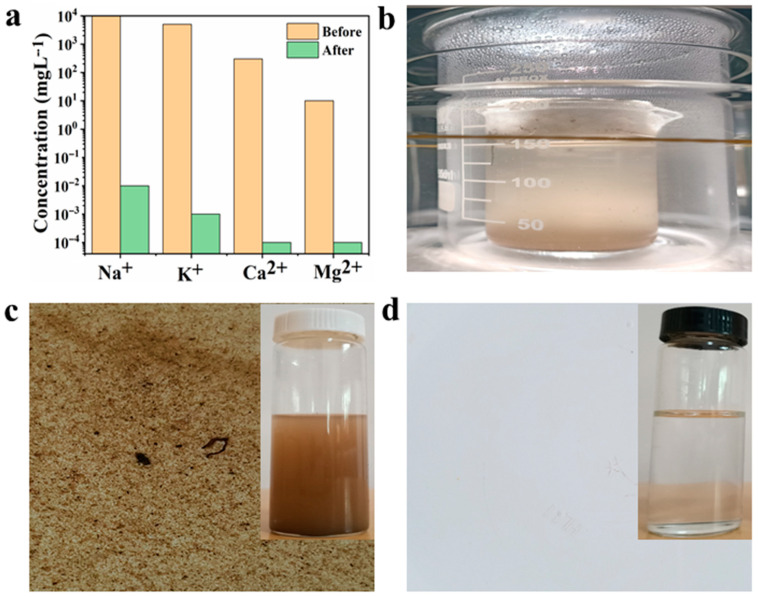
Salt rejection and water recovery via ACM solar evaporator: (**a**) ICP-OES spectroscopy profile of concentration gradient of different salt ions in simulated seawater, and condensed water; (**b**) real-time water condensing process from muddy wastewater; (**c**,**d**) optical morphology of muddy wastewater and ACM treated condensed water.

The ACM steam generator has the potential to effectively treat and purify the industrial and other wastewater. The impure industrial wastewater was collected from different resources. The designed system exhibits the potential for rejecting the salt ions as well as water recovery from different resources under natural sunlight intensity (1 kW m^−2^). We employed the Inductive coupled plasma atomic emission spectroscopy (ICP-OES) to inspect the four different salt ions concentration gradient (3.5 W% NaCl) within stimulated seawater and ACM treated condensed water. A comprehensive analysis of desalination process exhibits that the concentrations of Na^+^, K^+^, Ca^2+^, Mg^2+^ ions have been decreased remarkably after purifying it comparative to the amount of salt ions in the simulated seawater, as illustrated in Figure 6a. ACM matrix rejects the hazardous level of salt ion concentration by obtaining 99% purity and satisfy the World Health Organization (WHO) requirements for drinking water quality [32,33,34]. The desalination water meets the standard drinking water criteria by displaying the standard ingredients level of drinking water. Figure 6b represents the vapor condensation process from muddy wastewater and condensed water collection. According to WHO, statistics shows that 80% diseases spread by unsafe drinking water such as organic and inorganic toxins [4,35,36,37]. Mostly these toxins impurities are found in trace levels and many are responsible for the toxicity factors of the complex mixture, particularly wastewater, and are the principal cause of some of the world’s most dangerous bacterial infections. Comparatively, after passing through desalination process, rejection of salts ions and VOCs met the national drinking water Standard GB5749-85. Though various parameters such as TDS, COD, turbidity and so on that are in association with water purity can also be quantified as reported earlier [38]. A comparative analysis of both optical morphology of muddy wastewater and condensed water as illustrated in Figure 6c (before) and Figure 6d (after). Nonetheless, number modern salt rejection strategies can be applied for desalination of impure water as reported [39]. 

## 4. Conclusions

In summary, solar harvesting activated carbon decorated melamine (ACM) foam-based steam generating device is fabricated with enormous absorbing efficiency (92%) for efficient freshwater yield. Indeed, self-floatable steam evaporator holds strong potential for rejection against salt ions, volatile organic impurities, and muddy wastewater impurities. Open porous assembly of ACM allows smooth transport of water and localize the incident light and exhibits up to 80% photothermal conversion efficiency. As compared to other solar evaporating systems, ACM possesses outstanding flexibility (80% compressibility) without any material deterioration that enhances its portability and long-term endurance. The heat insulating ability of ACM is 10 times greater than pure water. A systematic, efficacious, and easily manufactured solar harvesting steam generation device that upholds the potential against a few wastewater impurities by supplying the single step freshwater production meeting the standard drinking water level.
