# Erratum: Arshad, N., et al. Super Hydrophilic Activated Carbon Decorated Nanopolymer Foam for Scalable, Energy Efficient Photothermal Steam Generation, as an Effective Desalination System. *Nanomaterials* 2020, *10*, 2510

**DOI:** 10.3390/nano11030676

**Published:** 2021-03-09

**Authors:** Naila Arshad, Iftikhar Ahmed, Muhammad Sultan Irshad, Hong Rong Li, Xianbao Wang, Shafiq Ahmad, Mohamed Sharaf, Muhammad Firdausi, Mazen Zaindin, Muhammad Atif

**Affiliations:** 1 Institute of Quantum Optics and Quantum Information, School of Physics, Xi’an Jiaotong University, Xi’an 710049, China; nailasehar371@gmail.com (N.A.); hrli@xjtu.edu.cn (H.R.L.); 2 Energy Research Centre, COMSATS University, Islamabad, Lahore Campus 54000, Pakistan; iftikhar@live.fr; 3 Ministry-of-Education Key Laboratory for the Green Preparation and Application of Functional Materials, Hubei Key Laboratory of Polymer Materials, School of Materials Science and Engineering, Hubei University, Wuhan 430062, China; sultan.danish93@gmail.com (M.S.I.); wangxb68@aliyun.com (X.W.); 4 Industrial Engineering Department, College of Engineering, King Saud University, P.O. Box 800, Riyadh 11421, Saudi Arabia; mfsharaf@ksu.edu.sa (M.S.); 438106660@student.ksu.edu.sa (M.F.); 5 Department of Statistics and Operations Research, College of Science, King Saud University, P.O. Box 800, Riyadh 11421, Saudi Arabia; zaindin@ksu.edu.sa; 6 Department of Physics and Astronomy, College of Science, King Saud University, Riyadh 11451, Saudi Arabia; muhatif@ksu.edu.sa

The authors wish to make the following corrections to this paper [1]:

The Funding and Acknowledgments sections need to be corrected. The updated information is provided below:

**Funding:** The team would like to express their thanks to the Deputyship for Research & Innovation, “Ministry of Education” in Saudi Arabia for financial support through the project number IFKSURG-1438-089. 

**Acknowledgments:** We thank technical facilitation offered by laboratories of Xianbao Wang and Hongrong Li for support in experimental work. Moreover, the team author extends their appreciation to the National Key R&D Program of China. 

This change does not affect the scientific results or conclusions in the original published paper.

The authors would like to apologize for any inconvenience caused to the readers by these changes.
